# Visuospatial working memory and attention control make the difference between experts, regulars and non-players of the videogame League of Legends

**DOI:** 10.3389/fnhum.2022.933331

**Published:** 2022-07-20

**Authors:** Carlos Valls-Serrano, Cristina De Francisco, María Vélez-Coto, Alfonso Caracuel

**Affiliations:** ^1^Department of Psychology, Catholic University of Murcia, Guadalupe, Spain; ^2^Department of Social Psychology, University of Seville, Seville, Spain; ^3^Mind, Brain and Behavior Research Center, University of Granada, Granada, Spain; ^4^Department of Methodology of Behavioral Sciences, University of Granada, Granada, Spain

**Keywords:** human-computer interaction, esports, working memory, attention, expertise

## Abstract

Video games have been postulated as an emerging field for studying the cognition-expertise relationship. Despite this, some methodological practices hinder scientific advance (e.g., heterogeneous samples, an ambiguous definition of expertise, etc.). League of Legends (LOL) is a massively played video game with a moderately defined structure that meets the requirements to overcome current study limitations. The aim of this study was to analyze cognitive differences among expert LOL players, regular LOL players, and non-videogame players. A sample of 80 participants was enrolled in three different groups of expertise. Participants were evaluated with behavioral tests of working memory, attention, cognitive flexibility, and inhibition. Kruskal-Wallis tests for group comparison showed that the experts performed significantly better than regular players and non-videogame players in the working memory test. Significant differences were also found between players and non-videogame players in the attention test. Methodological implications for future research in neuroscience and human-computer interaction are discussed.

## Introduction

The use of games to study human cognition has been present since the first steps of psychology as a scientific discipline. Some of the assessment tasks in the field were created even earlier, such as the Tower of Hanoi (Claus and Lucas, 1883). Originally designed as a mathematical game by Édouard Lucas in 1883, it is now one of the most widely used neuropsychological tests to assess executive functions (Hardy and Wright, 2018). However, the forerunner in this field was Binet (1894), who explored the cognitive processes involved in master chess players. In the following decades, many studies using chess have made it possible to study different cognitive topics. For instance, the relationships between cognitive constructs and chess skills (Burgoyne et al., 2016), the cognitive differences among chess players and non-chess players (Unterrainer et al., 2006), the architecture of the chess players brain (Hänggi et al., 2014) or the limits of human intelligence vs. artificial intelligence (Hassabis, 2017). In other words, chess has demonstrated that it is possible to investigate a large number of theoretical issues related to human cognition using a game.

Now, at the beginning of the twentieth century, the increasing popularity of video games around the world has postulated them as a new experimental paradigm for cognitive science (Gray, 2017). Video games are an emerging worldwide phenomenon with the impact of transforming human abilities, the culture, and the economy of our society. The number of video game players continues to grow year by year and is expected to reach 3.07 billion players in 2023 (Newzoo, 2020). The interest around video games is reflected in the increasing number of scientific publications in fields such as mental health (Paulus et al., 2018), gamification (Cheng, 2020), esports (competitive video game industry; Reitman et al., 2020), or cognitive sciences (Bediou et al., 2018), among others. According to the computer scientist and cognitive psychologist Allen Newell, video games are an excellent tool to explore “a genuine slab of human behavior” (Newell, 1973, p. 303). Several authors have endorsed this value of video games as a means of exploring human-computer interaction and expertise for a variety of reasons (Boot, 2015; Chabris, 2017; Campbell et al., 2018; Pluss et al., 2019): (i) Some video games are well-defined problems, with specific goals, clear start state, and stable transformation functions. These features allow researchers to analyze players’ execution in the same task over time; (ii) Most popular video games have large base players, allowing a wide range of low to high skilled players; (iii) They are implemented in a controlled setting where players are objectively tracked, increasing the reliability and validity of hundreds of data files available online and collected in real-time; (iv) Players carry out the activity in their everyday environment, far away from artificial settings that could condition participants’ responses. This allows the possibility to address aspects that cannot be easily assessed in the laboratory, for example, social interactions (Gray, 2017; Pluss et al., 2019).

Despite this promising approach, some authors warn that the continuous changes that have occurred in the last decades in the video game industry and players could affect the research methodology, making it obsolete (Dale et al., 2020). Two of the main problems of this field are the diversified profile of gamers (more generalized and less specialized) and the fuzzy boundaries between genres due to the increasing amount of hybrid games (Dale and Green, 2017). Video game genres (or, in other words, the characteristics of the problem that the players face) are a relevant aspect since they generally establish specific cognitive demands related to the game. Although the evolution of video games has blurred the limits among genres, there is enough evidence that action video games require higher attention or spatial demands than, for example, simulation games (Bediou et al., 2018; Sala et al., 2018). However, samples of gamers who play several genre games make it difficult to analyze how cognitive constructs are related with games that have similar and overlapping mechanics. On the other hand, investigations have used operational definitions to describe expertise that are far to be precise and do not represent a span of proficiency continuum. As a result, many studies mix different levels of expertise, confusing novice with naïve or experts with apprentices (Fiore et al., 2008). Finally, the criteria used to discriminate expertise levels have generally been based on time spent on video games (Latham et al., 2013). However, the evidence shows that deliberate practice on an activity is not directly associated with performance, so temporal criteria for delimiting expertise could not be appropriate (Macnamara et al., 2014).

In order to overcome these limitations, we consider the video game League of Legends (LOL) to be an optimal tool to study human cognition and expertise in the same way that chess was in the past. We argue two main reasons. Firstly, LOL is a moderately structured problem if it is compared with other video games (Goel and Grafman, 2000). This game takes place always on the same symmetrical game map, with the same goal, relatively stable rules, and transformation functions. This allows analysis of performance over time without changing structural conditions (usually found in other games). Second, LOL is a massive game played by millions of people around the world (Kollar, 2016). LOL has a general classification based on an ELO rating system like chess (Glickman and Jones, 1999), which makes it possible to distinguish expertise based on the ability of players in a more accurate way than other games.

For the moment, few studies have analyzed cognitive differences between LOL players, so the findings are scarce. Ding et al. (2018) found differences in the Multiple Object Tracking task among professional players, semiprofessional players, and casual players. Chang et al. (2017) found better performance in working memory and virtual-reality multitasking tests among LOL gamers and non-online players. Yao et al. (2020) also found that experts players had higher accuracy and larger working memory capacity in the visual change detection task. Qiu et al. (2018) found lower reaction times in LOL experts’ players than non-action video game players in the Useful Field of View. Finally, Li et al. (2020) found better cognitive flexibility and interference control in expert LOL players vs. regular players. Although these studies have interest in this field of knowledge, the criteria used to conceptualize the expertise are heterogeneous. A comparative resume about expertise criteria used on different LOL studies is provided in Table 1. Furthermore, little information about players profile is provided, as for example, the possible presence of Internet Gaming Disorder (IGD) or characteristics of the most played genre-games of participants. In summary, only one study analyzed differences between LOL experts and regular players and non-video game players (Ding et al., 2018) and the study of core executive components (attention control, inhibition, flexibility, and working memory) is limited, despite their relevance for initiating and monitoring goal-directed behavior in novel complex tasks (Friedman and Miyake, 2017) such as LOL. Despite the absence of specific studies, in the same line that other action videogames, we consider that playing LOL should require many cognitive constructs to deal with it (Pedraza-Ramirez et al., 2020). Action videogames players need to manage and manipulate a lot of information in the working memory (i.e., operate about abilities or items and their temporal information, calculate change about damage received or executed, etc.) (Yao et al., 2020). The top-down attention control is another crucial ability for players due to its relevance to maintain visual goals and avoid non-goal information interference (Föcker et al., 2019). Furthermore, in action videogames as LOL it is necessary to change between different tasks (e.g., control waves of neutral enemies, visit the map, gold information, etc.), so switching ability is of special importance (Li et al., 2020). Finally, inhibitory processes are involved practically during all the game, go/no go decisions are present in each fight or in any traced path (Li et al., 2020).

**TABLE 1:** Description of expertise criteria in LOL studies.

Study	Groups of participants	Expertise criteria
Chang et al. (2017)	MOBA players (*n* = 30). Players who reported only play LOL (*n* = 27). Non-online players (*n* = 58)	Players and non-players were classified based on The Internet Usage Questionnaire. Groups were formed based on the response of the following questions: Do you play any online game with interaction with other players? How many hours do you spend on playing such online game? Do you play any online game with single operation? How many hours? In the past year, how much time did you spend on internet a week?
Ding et al. (2018)	Professional players (*n* = 10) Semiprofessional players (*n* = 10) Casual players (*n* = 20)	LOL Secondary professional league Amateurs from a summer training camp. They shown potentials in LOL and might eventually become professional players. Casual players who self-reported playing the game frequently. 70% were classified in the in-game ranking.
Qiu et al. (2018)	Action video game experts (*n* = 15) Action video game non-experts (*n* = 14)	Two years of experience in action video games and expertise based on rank classification (percentile 93%) Less than 0.5 years of experience in action video games and rank classification between 29.92 and 45.11%.
Li et al. (2020)	Experts players (*n* = 35) Average players (*n* = 35)	Players ranked higher than Diamond tier (percentile 99.8%) Players ranked between Iron and Diamond tier
Yao et al. (2020)	Experts players (*n* = 18) Non-experts players (*n* = 19)	Two years of experience in action video games and expertise based on rank classification (percentile 93%) Less than 0.5 years of experience in action video games and rank classification between 29.92 and 45.11%.

*LOL, League of Legends; MOBA, Multiple Online Battle Arena.*

### Current study

Video game studies in the field of cognitive science need to overcome the methodology limitations of the last decades. LOL is one of the most popular video games in the world with massive base players. This makes it possible to objectively analyze the expertise of millions of players throughout the ELO ranking system. Furthermore, it has a moderately defined structure problem that helps to control the confounding variables present in other games like the changing objectives, rules, or scenarios. These conditions make this video game an ideal context for studying cognitive expertise in video games. The objective of this study was to analyze cognitive differences among expert LOL players, regular LOL players, and non-videogame players in a series of cognitive tasks focused on executive functions. Our hypothesis is that expert players will show a better performance than regular players, and the latter compared to the non-videogame players on these cognitive tasks.

### Methodology

### Participants

**FIGURE 1:**
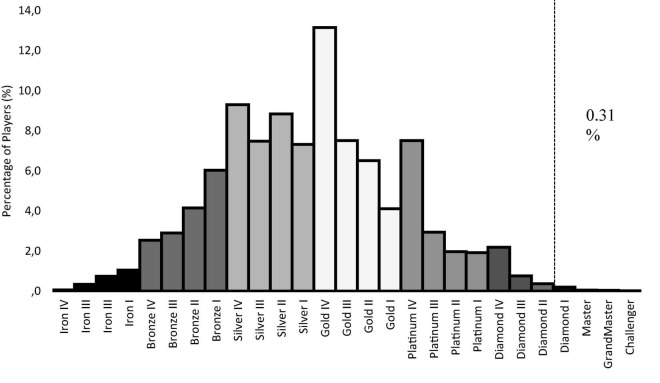
ELO ranking distribution League of Legends (season 9, 2019).

Eighty healthy young men were recruited for three groups of study participants: non-videogame players (*N* = 30), regular players (*N* = 30), and expert players (*N* = 20). Regulars and non-videogame players were recruited through a massive email announcement in the university. Experts were recruited from the UCAM esports club primary and secondary team that plays in the professional league in Spain. Only males were included in the study according to the recommendations of Pedraza-Ramirez et al. (2020). The inclusion criterion for non-videogame players was playing video games less than 1 h per week. Both groups of players met the two inclusion criteria of being (i) pure players of the MOBA genre, according to Dale and Green (2017) (participants had spent at least 2/3 of their time playing video games of the MOBA genre) and (ii) habitual players, according to Kokkinakis et al. (2017) (having played more than 100 LOL games in the last year). Expert players should also meet three demanding criteria. The first was (a) having been classified equal to or higher than Diamond I tier in the LOL ELO ranking system (distributed by tiers from iron to challenger). Players ranked beyond Diamond I are placed in a superior percentile than 99.69 (see Figure 1). This cut-off point placed these players above 2 standard deviations with respect to the mean in a sample of more than one million players (Milella, 2020). The second was being (b) a professional player from a primary or secondary professional LOL league. This last qualitative criterion was established in order to have more evidence that expert players have a proactive attitude toward peak performance. The exclusion criteria for all participants were: (i) suffering any disorder affecting their central nervous system, (ii) having consumed drugs in the last 48 h (except coffee and tobacco), and (iii) not exceeding the cuff-off score of 75 points on the Internet Gaming Disorder Test (IGD-20; Fuster et al., 2016).

### Instruments

The Corsi block tapping test (Kessels et al., 2008) from the computerized PEBL battery task (Mueller and Piper, 2014). It is a classic measure of visual spatial working memory. At the beginning of this test, nine fixed blue squares are shown in the screen, then a sequence is reproduced in which the squares are illuminated (1 s per square). The objective of the participants is to remember correctly the illuminated sequence. The start length sequence consists of two bright squares, and the longest sequence have nine bright squares. Each sequence has two trials and as long as the participant correctly remembers one of the two trials, he will go on to the next sequence, which will increase the length in one square. The task ends when two trials of the same length are incorrectly reproduced. This task consists of two conditions, forward condition, in which the sequence must be repeated in the same order, and backward condition, in which the sequence must be reproduced in reverse order. The outcome variables are the block span (the longest sequence correctly repeated under both conditions) and the correct trials (the sum of the number of trials correctly reproduced under both conditions).

The Antisaccade Task (AT; Roberts et al., 1994). It is part of the computerized battery task The Psychology Experiment Building Language (PEBL; Mueller and Piper, 2014). This task measures top-down attention control. During this task, participants are asked to focus their attention on an arrow shown in the center of the screen. Next, a square (for 225 ms) is shown on the left or right side of the screen (3.4 in from the cross), then another square is shown on the opposite side where the first square appeared. The second square contains the target stimulus, which remains on the screen for 150 ms before it disappears. The target stimulus is an arrow that can point in four directions (↑, ↓, ←, →). In this task, the participants are asked to try to ignore the first square and to pay attention to the second one, which includes the arrow. Then, participant has to indicate as quickly as possible the direction of the arrow. Before beginning the task, a practice trial is conducted with 22 stimuli; the final test consists of a total of 90 stimuli. The outcome variable is the mean percentage of correct responses and mean reaction time of correct responses.

The Number Letter Task (NLT; Miyake et al., 2000). The computerized version of the Inquisit 5 Lab battery (Inquisit, 2016) was applied. It is a classical task of cognitive switching. In this task, a 2 × 2 matrix is shown with a pair of characters (a digit and a letter, e.g., A7) that rotate in a predictive clockwise direction. When the pair of characters appears at the top of the matrix, the participants must respond by deciding if the letter is a consonant or a vowel. If the pair of characters appears at the bottom of the matrix, the participants must respond by deciding whether the number is even or odd. The task consists of three phases of practice, in which the task focused on the letter criterion (vowel or consonant) is tested (32 trials), another one focused on numbers (even or odd) (32 trials), and finally a practice of both previous conditions (16 trials). The final assessment task consists of a total of 128 trials, where both characters are shown with a random starting position. The outcome variables are the accuracy cost (difference between proportion correct) and the latency switch cost (difference between switch trials and non-switch trials).

The Stop Signal Task (SST; Verbruggen et al., 2019), a computerized task administered from the Inquisit 5 Lab battery (Inquisit, 2016) that measures response inhibition. During the task, participants observe in the center of the screen a circle where an arrow pointing to the left or to the right is displayed. The objective is to respond as quickly as possible according to the direction of the arrow. Although in some trials a sound could be presented after the appearance of the arrow, and under this circumstance the participants must inhibit any response. In each trial, the arrow is displayed for 2,000 ms. The initial stop signal delay between the arrow and the sound is 250 ms. But then the delay is modified according to hits (increase of 50 ms) and errors (decrease of 50 ms). The maximum delay between the appearance of the arrow and the stop signal is 1,150 ms, and the minimum is 50 ms. The test consists of a practice block of 32 trials, and the actual test consists of three blocks with 72 stimuli, of which a third are stop signal trials. The outcome variables are the probability of reacting in stop signal trials, the mean reaction time in correct non-signal trials, and the mean reaction time in stop signal trials (response of incorrect hits).

The Video Games Research Interview (VRI): An *ad hoc* semi-structured interview was developed based on a substance consumption precedent (Verdejo-García et al., 2005). Consumption data were recorded for the 10 most-consumed games over the last year, as well as the average of hours per day of games consumption and the number of days per week. Furthermore, for each game, the name, genre, platform played, average days and hours played per week, and number of years played were noted.

Internet Gaming Disorder Test (IGD-20) (Pontes et al., 2014; Spanish version of Fuster et al., 2016). It assesses the possible existence of an IGD using 20 items. A cutoff point of 75 (out of 100) was established for “disordered gamers” (Pontes et al., 2014).

### Procedure

Participants were evaluated between September 2019 and December 2020 in person in the UCAM psychology lab. All participants signed an informed consent form and were paid 10 euros for their participation. The study was carried out according to ethics for human research in the Declaration of Helsinki and was approved by the University Ethics Committee (CEO21906).

### Statistical analysis

First, descriptive analyzes were performed to describe the sociodemographic characteristics and the gambling profile of the participants. Second, the Kolmogorov-Smirnov test was used for testing normality, and the results recommended the use of non-parametric tests for independent groups. Third, we used the Kruskal-Wallis test for group comparison and *post hoc* analyzes were carried out to identify group differences. The dependent variables were the outcome variables described in the instruments section for each test, and the independent variable was the group (experts, regulars, or non-videogame players). The variables were inspected for extreme values and the participants were excluded for the respective test analysis. An outlier in the expert group was found in AT. Effect sizes for non-parametric tests were calculated according to Fritz et al. (2012). Fourth, Cohen’s d values were calculated for each of the significant differences between groups in index effect sizes. Fifth, we performed an extra analysis to deepen the results. The expert players group spent significantly more time playing LOL than regular players. We consider that this difference is a direct consequence of dedication to the competitive scene. But, to elucidate how time spent on video games is related to cognitive performance, we performed a Spearman correlation between time spent on LOL and the significant results found in the previous group comparisons. Furthermore, we divided all LOL players into two groups based on the time spent playing LOL and we used the median of this variable. Subsequently, we performed a group comparison using Mann-Whitney U. All statistical analyzes were performed using SPSS statistics v26.

## Results

**FIGURE 2:**
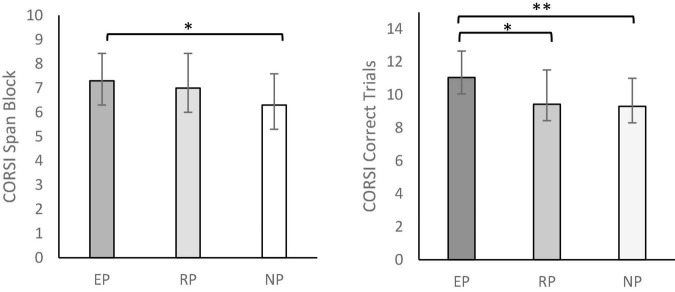
CORSI span block and correct trials performance. Error bars represents standard deviation. **(**EP, Experts Players Group; RP, Regular Players Group; NP, Non-videogame Players). *p < 0.05, ^**^p < 0.005.

**FIGURE 3:**
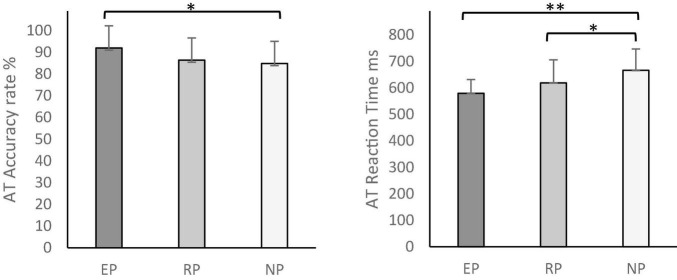
The Antisaccade Task (AT) accuracy rates (%) and reaction time (in ms). Error bars represents standard deviation (EP, Experts Players Group; RP, Regular Players Group; NP, Non-videogame Players). *p < 0.05, ^**^p < 0.005.

The sociodemographic scores for each group are shown in Table 2. Participants were matched in age and intelligence assessed with the Brief Kaufman Intelligence Test (Kaufman, 1990). But as expected, experts showed significantly less years of education than non-videogame players and more time spent playing video games than regulars. We consider both differences to be a direct consequence of dedication to the competitive scene; professional players usually report that they stop their academic studies due to the high temporal cost of training and competition. For this reason, we did not consider these variables as covariates in the statistical analysis. Cognitive performance in each test and group comparison analyses are provided in Table 3. Experts exhibit the best performance in all tests, followed by regulars and non-videogame players who had the worst performance. Significant differences between groups were found in the CORSI and AT tests. *Post hoc* analysis revealed that experts showed better span block results than non-videogame players in the CORSI test. Regarding correct trials in this test, experts also had better results than regulars and non-videogame players; no differences were found among these last two groups. With respect to AT, experts showed better accuracy than non-videogame players. Furthermore, experts and regulars displayed faster reaction time with respect to non-videogame players. Those significant results are illustrated in Figures 2, 3. No significant differences were found in the switching and inhibition tests.

**TABLE 2:** Sociodemographic and game features among groups.

	Experts mean (*SD*)	Regulars mean (*SD*)	Non-videogame players mean (*SD*)
Age	21.8 (2.2)	22.0 (3.2)	22.9 (3.5)
Years of education	18.7 (2.1)	19.1 (1.7)	20.1 (1.7)
Premorbid Non-verbal IQ	112.5 (6.9)	109.5 (8.6)	107.2 (8.3)
IGD-20: total score	50.2 (11.1)	45.6 (11.4)	30.8 (10.9)
VRI: Hours per week played to videogames	52.67 (19.55)	21.25 (15.39)	1.16 (3.06)
VRI: Hours per week played to LOL	45.3 (20.2)	17.4 (14.4)	0.5 (2.6)
LOL Season 9 (2019): Rank achieved	n (%)	n (%)	
Bronze	0 (0%)	4 (8%)	
Silver	0 (0%)	4 (8%)	
Gold	0 (0%)	13 (26%)	
<Diamond I	0 (0%)	9 (18%)	
Diamond I y Master	9 (18%)	0 (0%)	
Grandmaster	5 (10%)	0 (0%)	
Challenger	6 (12%)	0 (0%)	

*SD, Standard Deviation; IQ, Intelligence Quotient; IGD-20, Internet Gaming Disorder Test; VRI, Videogame Research Interview; LOL, League of Legends.*

**TABLE 3:** Cognitive performance differences among Experts Players (EP), Regular Players (RP), and Non-videogame Players (NP).

Test	Experts mean (*SD*)	Regulars mean (*SD*)	Non-videogame players mean (*SD*)	*H*	*p*	Cohen’s *d*	Group differences
CORSI block Span	7.3 (1.1)	7 (1.4)	6.3 (1.3)	7.288	0.026	0.54	EP > NP
CORSI correct trials	11.1 (1.6)	9.4 (2.1)	9.3 (1.7)	12.322	0.002	0.78	EP > (RP = NP)
AT accuracy rate (%)	91.9 (6.3)	86.4 (9.8)	84.9 (10.2)	7.896	0.019	0.58	EP > NP
AT reaction time (ms)	579.4 (52.0)	618.6 (87.1)	666.1 (80.4)	16.839	< 0.001	0.98	(EP = RP) > NP
NLT accuracy switch cost	−0.5 (0.0)	−0.5 (0.1)	−0.5 (0.0)	0.850	0.654		
NLT latency switch cost	550.9 (323.3)	586.6 (400.1)	645.7 (327.8)	1.744	0.418		
SST probability of reacting in stop signal trials	49.3 (6.4)	47.7 (4.4)	45.1 (9.2)	3.425	0.180		
SST reaction time in stop signal trials	475.3 (168.1)	496.5 (150.6)	517.9 (146.9)	1.636	0.441		
SST reaction time in non-signal trials	533.3 (197.0)	554.6 (182.2)	586.3 (191.6)	1.716	0.424		

*SD, Standard Deviation; ms, milliseconds; AT, The Antisaccade Task; NLT, The Number-Letter Task; SST, The Stop Signal Task.*

Finally, correlation analyzes between time spent on video games and CORSI and AT test were not significant for any variable [CORSI Block Span, *r* = 0.094, *p* = 0.517; CORSI Correct Trials, *r* = 0.225, *p* = 0.115; AT Accuracy Rate (%), *r* = 0.258, *p* = 0.071; AT Reaction Time (ms), *r* = −0.085, *p* = 0.557]. Group differences between LOL players who spent low time to video games and LOL players who spent high time to video games neither found significant results [CORSI Block Span, *U* = 293.5, *p* = 0.688; CORSI Correct Trials, *U* = 240.5, *p* = 0.156; AT Accuracy Rate (%), *U* = 214,5, *p* = 0.057; AT Reaction Time (ms), *U* = 282, *p* = 0.554].

## Discussion

The aim of this study was to investigate whether expertise in the video game LOL is associated with a superior cognitive ability compared to regular players and non-videogame players. We explored four main domains of executive functions related to goal-directed behavior (spatial working memory, attention control, switching, and inhibition) (Friedman and Miyake, 2017). The overall results showed that the experts had better spatial working memory and control attention. No significant differences were found for switching and inhibition abilities.

We used the Corsi Blocking Tapping test in order to assess spatial working memory ability. In terms of the spatial working memory ability, experts got a larger number of correct trials than regulars and those than non-videogame players. Regarding the spatial span, there were also significant differences among experts and non-videogame players. No significant differences were found among regulars and non-videogame players. Our findings support the results found by Yao et al. (2020), confirming that LOL requires managing and manipulating large amounts of information during a match, most of it visuospatial. Our results indicate that working memory would be important to carry out different cognitive processes such as control temporal information, spatial information, or mathematical calculations. Regarding other video game studies, our results are aligned with previous research that found an improvement in monitoring and updating of working memory tasks in action video game players (Colzato et al., 2013) or general video game players (Waris et al., 2019). Major evidence was supported in a meta-analysis that concluded better spatial cognition (including spatial working memory) in action video game players (Bediou et al., 2018). These results pointing to differences among experts and the other two both groups emphasize the importance of visual spatial working memory as a key feature in cognitive expertise.

Regarding the control attention, our results showed that experts and regulars exhibit shorter reaction times with respect to non-videogame players. Notwithstanding, the accuracy rates of experts was similar to regular and only superior to non-videogame players. These findings confirm that attentional control is a major process required at any point in the game, since players need to continuously maintain their visual goal and avoid non-goal information interference. These results are consistent with previous studies performed on LOL gamers that found better attentional performance in a variety of tasks. Ding et al. (2018) observed differences across professional players, regulars and non-videogame players using the multiple object-tracking task, but the lack of specific data prevents drawing clear conclusions about performance. It also has been proved the benefits on attention skills after 1-h LOL gaming session in non-videogame players, who showed an improvement on attention skills respect regulars (Qiu et al., 2018). In general, these results are aligned with previous literature that points that gamers have better attentional skills, although these advantages are more consistent in action video games players than non-action video games players (Kowal et al., 2018; Brodbeck and Dupuis, 2020). These results confirm that LOL as a game with action mechanics is not an exception and attentional processes are required to reach expert performance levels. In any case, results about reaction time and accuracy let us think that expert players could be more precise than non-videogame players, but their speed processing is similar to other players with lower levels of expertise.

LOL is a dynamic game that demands one to attend different tasks simultaneously, so switching ability should be a relevant cognitive process during a game. Players must focus on controlling the waves of neutral enemies while keeping opponents at bay without stopping to follow the map, items, gold information, etc. In our study, despite experts showing better performance than regular players and those respecting non-videogame players in the task-switching task, none of these differences did not reach significance. This lack of difference in switching was also found in a LOL study through a different task, the Flanker task (Ding et al., 2018). However, the Stroop task showed a lower switch cost in experts than in regular LOL players in the study by Li et al. (2020).

On the other hand, studies performed with other action-video game players found better performance respect non-videogame players in switch-cost tasks based on protocols for odd/even or smaller/larger categorizations, similar to the task we applied in our study (Green et al., 2012; Strobach et al., 2012). However, the main difference between those studies and ours was that they included a heterogeneous sample of action-video game players, so the differences among players’ profiles could explain this discrepancy. Despite this, Klaffehn et al. (2018) found similar task-switching performance among different genre players (shooter players, real-time strategy players, and non-videogame players) that dismiss the previous argument. Klaffehn et al. (2018) argue that differences in the assessment task applied could explain the mixed results found in the literature. It is known that the structure of the problem (i.e., the task) determines the cognitive demands (Goel and Grafman, 2000), but also, we consider that the assessment protocol could affect the interpretation of the results. Several studies have pointed out that apparently similar tests (including switching tests) contain subtle structural differences that could substantially change the cognitive components that are needed to succeed in the task (Bull et al., 2004; Li et al., 2019). In our opinion, switching ability is a process measured differently in some tasks, which is why the different results found in the literature. Furthermore, it has been shown that these tasks have a strong effect of practice on switching tasks (Strobach et al., 2012; Steyvers et al., 2019). Consequently, we consider that the absence of significant results could be explained not only by the novelty of the task for the participants, but also by the structural differences between the switching abilities involved in LOL and the test used in this study. For these reasons, we think that future investigations in video game players need support on consensual research protocols developed by experts, as has been done in other fields of knowledge (MacLean et al., 2018). Using a common framework could guide researchers about the features and measures that should be used, reducing the confusion derived from interpretation of findings.

Finally, we decided to test the inhibition control differences among our three groups of experts. Inhibitory control is a broadly studied construct in video game players, although mainly due to its relationship with addiction processes, specifically with the IGD (Argyriou et al., 2017). In any case, far from the field of psychopathology, inhibitory processes should be related to performance in video games. Most of them, including LOL, have game dynamics in which players need to force the cancelation of ab action. The results of our study found a higher probability to react to stop signals and a faster reaction time in stop signal trials in experts compared to regulars, and those with respect to non-videogame players, but these differences were not significant. The only study conducted on LOL players found lower false alarm rates and higher hit rates on a continuous performance test (Li et al., 2020). Due to these tests have notable structural differences regarding Stop Signal Task (SST) and comparison among them should be done with caution. Previous studies that used the SST in action video game players did not find significant differences compared to non- videogame players (Colzato et al., 2013; Steenbergen et al., 2015). Despite this, Deleuze et al. (2017) found differences among MOBA and shooter players, finding reduced abilities to cancel prepotent response in shooter players. Metcalf and Pammer (2014) also found a disinhibited performance in shooter players, but only in an addicted group. In the same direction a meta-analysis found a medium effect size [*d* = 0.56, 95% CI (0.32, 0.80)] comparing impaired response inhibition between IGD players and healthy players (Argyriou et al., 2017). In general, MOBA and shooter games are categorized as action video games, but these results confirm that similarities between these games are superficial while differences are more pronounced than they appear. Playing shooter video games needs to continuously attend stimulus and inhibit behavior to avoid shooting innocent players. Contrary to the shooter genre, LOL is a game in which players on the same team cannot harm each other, and therefore the demands of an inhibitory control are less demanding. Those evidence again point out the importance of controlling variables as the genre of games or analyze the possible existence of IGD participants in future studies. Furthermore, inhibition tasks are not the exception and have also been criticized for their concurrent and convergent validity of some inhibition tests (Paap et al., 2020; Raud et al., 2020). Using valid and reliable tests across studies should be a priority for easy comparison and interpretation of studies.

Finally, when we gather all LOL players, we did not find any correlation between time spent on LOL and attention control inhibition and spatial working memory. Likewise, we did not find differences on these cognitive constructs between LOL players who spent higher time on LOL and players who spent lower time on LOL. These results support the idea that better performance on attention control inhibition and spatial working memory in experts’ players cannot be explained only by a deliberate practice. This idea is aligned with several studies that analyzed expertise in different domains (e.g., games, sports, music, education, or professions) and concluded that practice is important, but not as important as has been pointed (Macnamara et al., 2014).

It is important to note that this study presents some limitations that must be considered. Although this research is an exploratory study, the sample is small but similar to that of the rest of the works in this field. Although we think that differences in years of education are a direct consequence of dedicating yourself professionally to esports competition, experts had an important difference in cognitive performance with respect to other groups. To find pure-genre LOL players, the cut-off established by Dale and Green (2017) (2/3 of their video game time) was applied, but even that allows the influence of other video game practices. In addition, the influence of other cognitive activities than video games were not controlled. The VRI used in the study is a tool that was not validated previously in videogame users. Finally, it is important to point out that expertise in LOL depends on tens of variables (e.g., mechanics, metagame, group communication…) (Donaldson, 2017; Pedraza-Ramirez et al., 2020), in any moment these results should be considered as the only factors related to performance.

This study provides a step forward for the studies developed in the field of cognitive psychology and human-computer interaction with a controlled methodology of sample characteristics and study design. We insist accordingly with Dale and Green (2017) that the methodology of video game studies should be more precise. Consequently, future studies should be more rigorous selecting the profile of players, analyzing pure-genre players, and if possible, using well-structured single games. Furthermore, comparisons among studies should consider in detail the study design and tasks used to avoid existing confusion in some results and be more precise with interpretation of the data. In the same direction, some cognitive abilities are highly task dependent; and for this reason, it would be interesting to explore cognitive performance using an in-game context. Finally, these results could have implications in other fields such as sports psychology. Our results suggest that the cognitive benefits of playing video games have a limit in relation to experience. For this reason, training programs focused on working memory abilities could enhance gaming performance on regular players, and hence facilitate reach expertise achievement.

In conclusion, this study shows that experts’ pure genre LOL players can be distinguished by a high performance in visual spatial working memory test respect to regular players and non-videogame players. Independently of the level of expertise, LOL players also showed better results than non- videogame players on the attentional control test. On the other hand, no differences were found among the groups in switching ability and inhibition processes.

## Data Availability Statement

The datasets presented in this study can be found in online repositories. The names of the repository/repositories and accession number(s) can be found below: https://osf.io/4mxh2/?view_only=219e2ae95a864fb088dbb618464de16e.

## Ethics statement

The study was carried out according to ethics for human research in the Declaration of Helsinki and was approved by the University Ethics Committee (CE021906). The patients/participants provided their written informed consent to participate in this study.

## Author contributions

CV-S and AC contributed to conception and design of the study. MV-C organized the database. CD performed the statistical analysis. CV-S wrote the first draft of the manuscript. CV-S, AC, MV-C, and CD wrote sections of the manuscript. All authors contributed to manuscript revision, read, and approved the submitted version.

## Conflict of Interest

The authors declare that the research was conducted in the absence of any commercial or financial relationships that could be construed as a potential conflict of interest.

## Publisher’s Note

All claims expressed in this article are solely those of the authors and do not necessarily represent those of their affiliated organizations, or those of the publisher, the editors and the reviewers. Any product that may be evaluated in this article, or claim that may be made by its manufacturer, is not guaranteed or endorsed by the publisher.
